# A population-based study on prevalence and predisposing risk factors of infant functional gastrointestinal disorders in a single center in Southern Fujian

**DOI:** 10.3389/fped.2022.993032

**Published:** 2022-09-29

**Authors:** Huanhuan Huang, Caiyun Wang, Wei Lin, Yongbin Zeng, Bin Wu

**Affiliations:** ^1^Department of Pediatrics, The First Affiliated Hospital, Fujian Medical University, Fuzhou, China; ^2^Department of Pediatrics, Jinjiang Maternity and Child Health Hospital, Quanzhou, China; ^3^Department of Laboratory Medicine, Gene Diagnosis Research Center, The First Affiliated Hospital, Fujian Medical University, Fuzhou, China; ^4^Fujian Key Laboratory of Laboratory Medicine, The First Affiliated Hospital, Fujian Medical University, Fuzhou, China

**Keywords:** infant, functional gastrointestinal disorders, regurgitation, colic, constipation

## Abstract

**Background and aim:**

The prevalence of infant functional gastrointestinal disorders (FGIDs) varies across different areas but is largely unknown in southern Fujian. The aim of this study is to evaluate the prevalence of infant FGIDs in southern Fujian according to Rome IV diagnostic criteria.

**Methods:**

A cross-sectional prospective questionnaire-based survey was conducted among healthy infants between 0 and 3 months of age in southern Fujian. A total of 1,006 infants who received a physical examination from October 2017 to October 2018 were recruited in this study. Parents or caregivers provided demographic information and completed the questionnaire on gastrointestinal symptoms for infants. Infants with FGIDs were diagnosed using the Rome IV criteria.

**Results:**

Based on the Rome IV criteria, the prevalence of having a FGID in infants is 58.3% (586/1,006). The most common FGIDs in infants were regurgitation (45.7%, 460/1,006), followed by difficult defecation (3.6%, 36/1,006), functional constipation (3.2%, 32/1,006), and colic (2.4%, 24/1,006). No infants fulfilled diagnostic criteria for rumination syndrome and cyclic vomiting syndrome. Among the infants with FGIDs, 457 cases (78.0%, 457/586) were found with single FGID. Combined FGIDs were diagnosed in 129 (22.0%, 129/586) infants; of whom, 21.2% (124/586) had double disorders and 0.9% (5/586) had triple disorders. The most common combined FGIDs were regurgitation and difficult defecation (12.8%), followed by regurgitation and colic (2.4%). Risk factor analysis revealed that younger paternal age (*B* = 0.424, *P* = 0.004), paternal history of FGIDs (*B* = 0.821, *P* = 0.000), maternal history of FGIDs (*B* = 0.427, *P* = 0.012), and probiotics received in infant (*B* = 0.324, *P* = 0.032) were associated with an increased risk of infant FGIDs, whereas vitamin D supplementation after birth (*B* = −0.690, *P* = 0.000) can reduce the risk of developing FGIDs.

**Conclusion:**

FGIDs are common in infants living in southern Fujian according to Rome IV diagnostic criteria. The most common FGIDs in infants were regurgitation, difficult defecation, and functional constipation. Factors including younger paternal age, parental history of FGIDs, and the probiotic supplementation in infant showed a significant association with infant FGIDs. Whereas, vitamin D supplementation in infant was found to be a protective factor against FGIDs.

## Introduction

Functional gastrointestinal disorders (FGIDs) are chronic or recurrent symptoms that cannot be explained by structural or pathologic abnormality. Infants have a relatively immature gastrointestinal function, which makes them more likely to have a variety of FGIDs (1). Infant regurgitation (IR), infant colic (IC), rumination syndrome (RS), infant dyschezia (ID), functional diarrhea (FD), functional constipation (FC), cyclic vomiting syndrome (CVS) are the main diagnosis of FGIDs in infants according to Rome IV criteria (2). The prevalence of FGIDs infants has a wide range which has been reported from <1% to as high as 83%(3, 4). One of the reasons for the prevalence difference may be due to different diagnostic criteria used, different study respondents, and different geographical location in different studies (1, 5). FGIDs do not only have negative impacts on the physical growth and quality of life of affected infants, but also on the emotional status and daily life of the family (6, 7). Parents may have less ability to concentrate at work and feel anxious, and even seek to consult a physician frequently, which will not only increase medical costs, but also lead to needless suffering of the caregivers (8, 9).Therefore, investigating the prevalence and risk factors of FGIDs in infants are of great clinical importance.

Due to the lack of biological or physiological markers, the investigation of FGIDs in infants is usually performed by using questionnaires completed by parents or caregivers. Rome IV criteria is the latest diagnostic criteria for infant FGIDs (2). However, there is still limited report on the using the criteria for clinical epidemiological investigation of infant FGIDs in China, especially in southern Fujian (10, 11). Meanwhile, epidemiological data on FGIDs in infants remain controversial (12). The current study aims at evaluating the prevalence and the predisposing risk factors of FGIDs in infants aged 0 to 3 months and followed-up to 1 year of age in a sample of southern Fujian using Rome IV criteria.

## Materials and methods

### Study population

Infants aged 0 to 3 months who were examined and followed up regularly in Jinjiang Maternity and Child Health Hospital were included in this study. The infants of the sample by stratified sampling based on the reported child health care population data in Jinjiang City, Fujian Province in 2017. The data were obtained from 19 townships in Jinjiang City (Supplementary Table 1). Our sampling covered the whole area of Jinjiang City. Besides, they were able to be followed-up to 1 year of age.

The inclusion criteria for all participants were as follows: (1) Infants 0–3 months of age. (2) Physical examinations performed had been found negative and fully recorded in Jinjiang Maternal and Child Health Hospital. (3) Parents or caregivers had been informed about the study and signed a consent form. (4) Parents or caregivers were able to cooperate with the regular follow-up. (5) No gastrointestinal treatment drugs were used.

The exclusion criteria for all participants were as follows: (1) Incomplete physical examination data. (2) Organic diseases or acute infections. (3) Unable to regular follow-up. (4) The infants in need of probiotics to treat diseases.

This study was conducted from October 1, 2017, to October 30, 2018, in Jinjiang Maternal and Child Health Hospital. Written informed consent was obtained from the parents or caregivers after providing details of the survey.

### Methods

The detailed medical history including physical examination, and basic and auxiliary examinations were done by pediatricians. Based on the examination results, the diagnosis will be made according to Rome IV criteria. The questionnaire consisted of three parts: Part 1 contained information on demographics characteristics, and gastrointestinal symptoms including regurgitation, colic, difficulty defecation, constipation etc. Part 2 contained infants' growth and development indexes. Part 3 collected information on the predisposing risk factors for infant FGIDs. The detailed questionnaire was provided in the Appendix 1.

The enrolled infants were followed up every 3 months till 1 year of age. The medical history, physical examination, basic and auxiliary examinations, and questionnaire were done for each follow-up. The result of the last follow-up (1 year of age) was used to determine whether the infant's symptoms met the Rome IV criteria. Body weight, body length and laboratory data such blood routine examination, fecal routine examination, occult blood, and 25-hydroxyvitamin D level were obtained at 6 months and 12 months old, respectively.

### Statistical analysis

Data were entered into Epidata (version 3.1, Odense, Denmark). The statistical analysis was performed using statistical analysis software SPSS package version 23.0 for Mac OS X (SPSS Inc., Chicago, IL). A descriptive statistic was used to present the characteristics of the patients and the prevalence rates FGIDs after diagnosis modification. Continuous variables were expressed as means ± standard deviations. *Chi-squared*, nonparametric and *Student's t*-test were performed to compare categorical and continuous variables between groups. Univariate and multiple logistic regression were used to identify risk factors associated with the development of FGIDs (such as demographic characteristics, social- economic factors, adverse events exposure). Odds ratio (OR) estimates and 95% confidence interval of OR, as well as the *P*-values of the *Wald chi-square* test for each risk factor were provided. A *P* < 0.05 was considered statistically significant. Multiple logistic regression was performed on variables that were found to have significant associations.

## Results

### Clinical characteristics of the enrolled infants

A total of 1,086 individuals agreed to participate in this study from October 2017 to October 2018. The infants were not known to have any FGIDs prior to administration of the questionnaire. Sixty infants were lost to follow-up during the study period. Twenty infants were excluded due to the diagnosis of other diseases during follow-up. Consequently, finally 1,006 cases were included in our study. The age of the enrolled infants was 0 to 3 months old, with an average age of 1.79 ± 1.77 months.

### Infant FGIDs diagnosis modification during regular follow-up visits

In this study, the initial diagnosis was revised at each visit based on symptoms, ancillary investigations, and response to treatment. To compare the initial diagnosis and final diagnosis, we defined: diagnosis modification rate = the number of cases with discordant initial and final diagnosis / total number of cases (*N* = 1,006). After continuous long-term follow-ups, the diagnosis was modified in 4.8% of the total 1,006 enrolled infants. IR was the most likely symptom to experienced diagnostic modification, followed by FD. The second (6-month-old) and third (9-month-old) follow-up visits were the mostly likely to undergo diagnosis modification (Tables 1, 2).

**Table 1:** Infant FGIDs diagnosis modification rate during regular follow-up visits.

**FGIDs**	**3-month follow-up,** ***n* (%)**	**6-month follow-up,** ***n* (%)**	**9-month follow-up,** ***n* (%)**	**12-month follow-up,** ***n* (%)**	**Total** ***n* (%)**
IR	7 (0.7)	9 (0.9)	0 (0.0)	0 (0.0)	16 (1.6)
IC	1 (0.1)	2 (0.2)	0 (0.0)	0 (0.0)	3 (0.3)
ID	0 (0.0)	4 (0.4)	4 (0.4)	0 (0.0)	8 (0.8)
FD	0 (0.0)	0 (0.0)	9 (0.9)	7 (0.7)	16 (1.6)
FC	1 (0.1)	0 (0.0)	4 (0.4)	0 (0.0)	5 (0.5)
Total	9 (0.9)	15 (1.5)	17 (1.7)	7 (0.7)	48 (4.8)

IR, infant regurgitation; IC, infant colic; ID, infant dyschezia; FD, functional diarrhea; FC, functional constipation.

**Table 2:** The detailed data of infant FGIDs diagnosis modification rate.

**Final diagnosis**	**Initial diagnosis**	**Number of cases (%)**
IR (*n* = 16)	Improper feeding practices	8 (50.0)
	Spilled milk^a^	5 (31.2)
	Diagnosis unknown	3 (18.8)
IC (*n* = 3)	Improper feeding practices	3 (100.0)
ID (*n* = 8)	Changes in feeding practices	2 (25.0)
	Diagnosis unknown	6 (75.0)
FD (*n* = 16)	Dyspepsia	13 (81.2)
	Diagnosis unknown	3 (18.8)
FC (*n* = 5)	ID	5 (100.0)

IR, infant regurgitation; IC, infant colic; ID, infant dyschezia; FD, functional diarrhea; FC, functional constipation.

a Regurgitation time does not meet Rome IV criteria.

### Prevalence of FGIDs in infants

**Figure 1:**
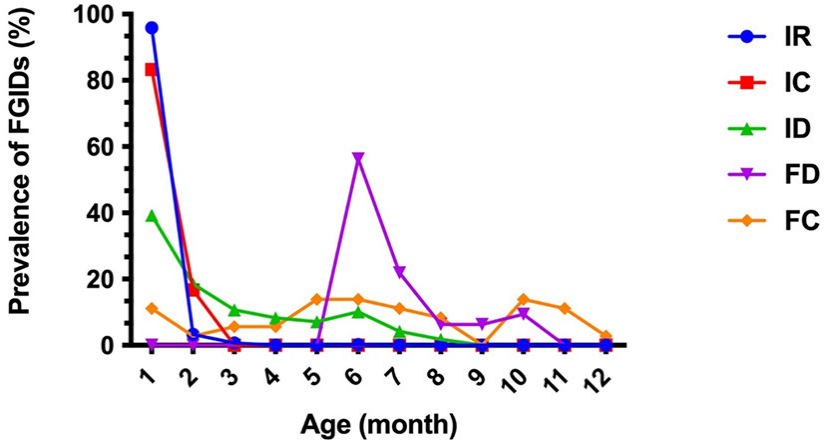
Trend of prevalence of FGIDs in infants aged from 1 to 12 months old. IR, infant regurgitation; IC, infant colic; ID, infant dyschezia; FD, functional diarrhea; FC, functional constipation.

Of the 1,006 enrolled infants, 58.6% (586/1,006) of these developed FGIDs; of whom, 460 individuals (45.7%) had IR, 168 individuals (16.7%) had ID, 36 individuals (3.6%) had FC symptom, 32 individuals (3.2%) had FD and 24 individuals (2.4%) had IC. No rumination syndrome (RS) and cyclic vomiting syndrome (CVS) were found in the study. A total of 457 individuals (78.0%, 457/586) were found with single FGID, 124 individuals (21.2%, 124/586) coexisted with two FGIDs, five individuals (0.9%, 5/586) coexisted with three FGIDs (Table 3). No individual was found with four or more FGIDs. The trend of prevalence of FGIDs in infants aged from 1 to 12 months old was shown in Figure 1. IR and IC mostly occurred within 1 month of age (95.9%, 83.3%), gradually reduced with age, and almost no longer occurred from 3 months of age. ID also had the highest incidence (39.3%) in 1 month of age, while it declined slowly with age. Besides, it was basically stable at about 10% from March to June and rare in September. Notably, the incidence of FD occurred was higher after 6 months of age (56.3%), and then gradually improved frequently until 11 months of age. FC covered the period from 1 to 12 months of age.

**Table 3:** Prevalence of common functional gastrointestinal disorders in infants aged from 1 to 12 months old.

**Type of disorders**	***n***	***%***
**Single FGID**	**457**	**78.0**
IR	343	58.5
IC	5	0.9
ID	78	13.3
FD	17	2.9
FC	14	2.4
**Double FGIDs**	**124**	**21.2**
IR+IC	14	2.4
IR+ID	75	12.8
IR+FD	12	2.0
IR+FC	11	1.9
IC+ID	1	0.2
IC+FD	1	0.2
IC+FC	1	0.2
ID+FD	1	0.2
ID+FC	8	1.4
**Triple FGIDs**	**5**	**0.8**
IR+IC+ID	2	0.3
IR+ID+ FD	1	0.2
IR+ID+FC	2	0.3

IR, infant regurgitation; IC, infant colic; ID, infant dyschezia; FD, functional diarrhea; FC, functional constipation. Bold numbers represent the total number or total percent.

### Risk factors of infant FGIDs

The potential causal factors that related to the occurrence of FGIDs in infants, such as pregnancy history, infant birth history, feeding history, growth and development history, family living environment and socioeconomic status, etc. were analyzed. Univariate analysis of risk factors for above-mentioned factors showed that birthplace, paternal age, paternal history of FGIDs, maternal history of FGIDs during pregnancy, vitamin D supplementation in infant after birth, and the probiotic supplementation in infant after birth were significantly associated with an increased risk of FGIDs (Supplementary Table 2).

Multivariate analysis showed that younger paternal age, parental history of FGIDs and the probiotic supplementation in infant, were significantly more likely to have FGIDs, while vitamin D supplementation in infant remained significantly associated with lower risk of FGIDs (Table 4).

**Table 4:** The results of multiple factor unconditional logistic regression analysis on infant FGIDs.

**Risk factor**	***B***	***SE***	***Wald***	***P***	***OR (95%CI)***
Paternal age	0.424	0.147	8.286	0.004	1.528 (1.145 2.040)
Father with FGIDs history	0.821	0.166	24.451	0.000	2.273 (1.642 3.148)
Mother with FGIDs history	0.427	0.171	6.239	0.012	1.533 (1.096 2.143)
Probiotics supplementation^a^	0.324	0.152	4.584	0.032	1.383 (1.028 1.862)
Vitamin D supplementation	−0.690	0.193	12.832	0.000	0.502 (0.344 0.732)

B, regression coefficients; SE, standard error; Wald, Chi-square value; OR, odds ratio.

a Only infants on long-term (more than 3 weeks) probiotic supplementation were included in the study. Supplemental probiotics including Lactobacillushelveticus, Lactobacillus rhamnosus and Reuteri DSM 17938.

### The growth and development indexes of infants during follow-up

The growth and development indexes of infants during follow-up were shown in Table 5. However, there was no statistically significant difference in weight, height, hemoglobin level, and vitamin D level between groups.

**Table 5:** Comparison of the weight, height, hemoglobin level, and vitamin D levels of infants at month 6 and month 12.

**Indicator**	**Gender**	**Number of cases**	**Infants with FGIDs**		**Infants without FGIDs**		***t***	***P***
			***n***	$\bar{x}$ **±s**	***n***	$\bar{x}$ **±s**		
**Month 6**								
Weight (kg)	male	550	311	8.3 ± 0.9	239	8.3 ± 0.9	−0.038	0.970
	female	456	271	7.6 ± 0.9	185	7.6 ± 1.0	0.433	0.666
Height (cm)	male	550	311	68.1 ± 2.4	239	68.1 ± 2.2	0.129	0.898
	female	456	271	66.3 ± 2.0	185	66.4 ± 2.3	−0.520	0.603
Hemoglobin (g/L)		1,006	582	115.7 ± 8.5	424	115.2 ± 8.8	1.001	0.317
Vitamin D (ng/ml)		1,006	530	30.4 ± 5.4	476	30.3 ± 5.2	1.390	0.443
**Month 12**								
Weight (kg)	male	550	311	10.0 ± 1.1	239	10.0 ± 1.1	0.131	0.896
	female	456	271	9.3 ± 0.9	185	9.2 ± 0.9	1.300	0.194
Height (cm)	male	550	311	76.1 ± 2.5	239	76.0 ± 2.3	0.594	0.553
	female	456	271	74.4 ± 2.1	185	74.5 ± 2.3	−0.595	0.552
Hemoglobin (g/L)		1,006	582	118.4 ± 8.7	424	118.6 ± 9.2	−0.425	0.671
Vitamin D (ng/ml)		1,006	530	31.4 ± 8.9	276	30.4 ± 9.2	1.090	0.276

## Discussion

Prevalence of and risk factors for FGIDs in infants from 0 to 12 months are still scarce and remain controversial (5). To the best of our knowledge, our study is the first to investigate the epidemiology and risk factors regarding FGIDs in infants in southern Fujian using Rome IV criteria.

Among the 1,006 infants enrolled in the study, 586 infants were diagnosed with FGIDs, with a prevalence of 58%, which was different from previous reports (4, 6, 12–14). In general, the difference may be related to cultural influences in the perception of symptom severity in addition to genetic or feeding differences, and the different diagnostic criteria used (1). It should be noted that the prevalence in our study with modified diagnosis tended to more accurate compared with previous studies (15). In current study, the diagnosis modification rate of only a single questionnaire investigation is about 5%, and the diagnosis can be modified once or twice more through continuous follow-up visits, which suggests that multiple diagnostic corrections is required in the diagnosis of infant FGIDs.

IR is the most common FGID across the world in infants and young children (13, 16, 17). The current study also found the prevalence of IR was 45.7%, which mainly occurred within 1 month of age (95.9%). The prevalence IR of our results was much higher compared with previous studies which also used the Rome IV criteria (4, 13). Infants with ID were also common infant FGIDs, with a prevalence of 16.7%, which was consistent with previously reported meta-analysis data of larger cohorts with a wide range from 0.5 to 32.2% (18). The prevalence of FC, FD, and IC accounted reported in this study was < 5% (3.6, 3.2, and 2.4%), which was in broad agreement with a previous study conducted in China (14). Moreover, we found 22.0% of infants having more than one FGID, of which IC and IR was the most find combination. However, no RS and CVS were found in this study. This could be due to the lower incidence of rumination and cyclic vomiting in infants and the difficultly for parents or caregivers to identify these syndromes. From the present study (Figure 1), it is apparent that FGIDs is a dynamic process, thus it is necessary for us to monitor the changes in gastrointestinal symptoms across time to capture meaningful dynamic changes.

In this study, we found a significant association between younger paternal age and the occurrence of FGIDs in infants. The exact reason is still unclear. However, based on the studies reported in the literature, it is tempting to speculate that on the one hand, father with younger age may have an immature mentality and lack the knowledge and experience with childcare. On the other hand, lower childbearing age may indirectly reflect the poor family economic conditions, which influences the likelihood that an infant will be taken good care of (19, 20).

Our results indicated that paternal history of FGIDs was also associated with an increased risk of infant FGIDs. Although no genes affecting the occurrence of FGIDs have been identified so far, genetic background is still an important factor in susceptibility to FGIDs. The same family living environment, eating habits, and social living environment mean that there are the same disease susceptibility factors. In addition, if their parents have a history of FGIDs, the infants have a tend to have the manifestations of FGIDs than those without. In contrast, Chogle et al. reported that the family history of FGIDs did not influence the prevalence of FGIDs in infants (17). Consequently, the association between genetic background and FGIDs was suspicious, and it still needed further evaluation.

Researchers have focused a lot of attention on the use of probiotics as a treatment or prevention for gastrointestinal disorders. However, the potential beneficial effects of probiotics on infant FGIDs are still controversial. Capozza et al. have found some specific probiotics showed a significant reduction in crying and fussing compared to placebo in breastfed infants with colic. Besides, in irritable bowel syndrome (IBS), constipation and gastroesophageal reflux, specific strains of probiotics can improve the related symptoms, although current evidence is insufficient to provide any specific recommendation for the prevention or treatment of the related FGIDs (21). However, according to a study performed by Mantaring et al., probiotics supplementation may not improve infants' gastrointestinal symptoms (22). In this study, no benefit has also been demonstrated for probiotic supplementation in infant, or even it may be one of the risk factors for infants to develop FGIDs. Despite that, parents are easier to choose supplemental probiotics due to gastrointestinal symptoms developed in infants or due to taking advice from other breastfeeding mothers.

Vitamin D is an important micronutrient that plays a critical biological role in various processes in human tissues (23). The risk of vitamin D deficiency occurs at all stages of life, including during pregnancy, in infants and children (24). It is really a fact that vitamin D supplementation is beneficial to the treatment of many gastrointestinal diseases (25). In our study, we found an interesting phenomenon that vitamin D supplementation is associated with a lower risk of FGIDs while its levels didn't show significant difference between infants with and without FGIDs. Such finding might be attributable to the fact that vitamin D supplementation could reduce the prevalence of FGIDs in infants by some yet unknown mechanism to be further investigated, while serum vitamin D is an outcome variable at 6 and 12 months of age, which may be affected by numerous factors such as the duration of light exposure, unbalance diet, and digestive absorption, etc. in infants (26, 27). Thus, the results deserve more in-depth research in the future. *American Academy of Pediatrics Guidelines* stated that “breastfed and partially breastfed infants should be supplemented with 400 IU/day of vitamin D beginning in the first few days of life,” and that “supplementation should be continued unless the infant is weaned to at least 1 L/day or 1 qt/day of vitamin D-fortified formula or whole milk.” It was also stated that “All non-breastfed infants, as well as older children who are ingesting < 1,000 ml/day of vitamin D-fortified formula or milk, should receive a vitamin D supplement of 400 IU/day” (28). Consequently, personalized supplementation with vitamin D is necessary in infants.

In summary, the current findings suggest that the prevalence of FGIDs are high in infants living in Jinjiang city. We found that the common FGIDs in infants were regurgitation, difficult defecation, and functional constipation. Factors such as younger paternal age, parental history of gastrointestinal dysfunction, and the supplementation of probiotics after birth showed a significant association with infant FGIDs. Whereas, vitamin D supplementation was found to be a protective factor for infant FGIDs. The limitation of current study is that all infants were obtained from a single center, even though this hospital is one of the largest maternal and child health hospitals that receive FGID infants from all over southern Fujian. Multicenter studies are still needed in the future.

## Data availability statement

The original contributions presented in the study are included in the article/Supplementary material, further inquiries can be directed to the corresponding authors.

## Ethics statement

The study was approved by Ethics Review from Branch from Research and Clinical Technology Application, Ethics Committee of Jinjiang Maternal and Child Health Hospital, and carried out according to the 1975 Declaration of Helsinki. Written informed consent was obtained from the study subjects before their enrolment.

## Author contributions

YZ and HH: data analysis and manuscript writing. HH and CW: data collection. YZ, WL, and BW: project development and manuscript editing. All authors critically reviewed the manuscript. All authors read and approved the final manuscript.

## Funding

The study was supported by a grant from National Natural Science Foundation of China (81601834).

## Conflict of interest

The authors declare that the research was conducted in the absence of any commercial or financial relationships that could be construed as a potential conflict of interest.

## Publisher's note

All claims expressed in this article are solely those of the authors and do not necessarily represent those of their affiliated organizations, or those of the publisher, the editors and the reviewers. Any product that may be evaluated in this article, or claim that may be made by its manufacturer, is not guaranteed or endorsed by the publisher.
