# Supplementary material for: A population-based study on prevalence and predisposing risk factors of infant functional gastrointestinal disorders in a single center in Southern Fujian
Source: Front Pediatr. 2022 Sep 29;10:993032. doi: 10.3389/fped.2022.993032 (PMC9557738; doi:10.3389/fped.2022.993032)
Supplement: Supplementary file 1 [file Table_1.docx]

**Table S1** The number of live births and allocation cases by stratified sampling

from 19 townships in Jinjiang City, Fujian Province in 2017

| \| Township \| \| --- \| | Number of live births | Allocation cases |
| --- | --- | --- | --- |
| Xin Tang | 680 | 41 |
| Mei Ling | 757 | 45 |
| Ling Yuan | 608 | 37 |
| Qing Yang | 919 | 45 |
| Luo Shan | 777 | 46 |
| Xi Yuan | 632 | 38 |
| Ci Zao | 1473 | 83 |
| Jin Jing | 812 | 47 |
| Ying ling | 883 | 52 |
| Chen Dai | 1671 | 93 |
| Long Hu | 1271 | 72 |
| Dong Shi | 1620 | 90 |
| An Hai | 2203 | 121 |
| Yong He | 1264 | 74 |
| Chi Dian | 1322 | 75 |
| Shen Hu | 636 | 39 |
| Nei Keng | 1062 | 61 |
| Zi Mao | 230 | 17 |
| Xi Bin | 92 | 10 |
| Total | 18912 | 1086 |
