# Supplementary material for: A population-based study on prevalence and predisposing risk factors of infant functional gastrointestinal disorders in a single center in Southern Fujian
Source: Front Pediatr. 2022 Sep 29;10:993032. doi: 10.3389/fped.2022.993032 (PMC9557738; doi:10.3389/fped.2022.993032)
Supplement: Supplementary file 2 [file Table_2.docx]

**Table S2** Univariate analyses of risk factors associated with FGIDs

| Variables | Stratification variables | Infants without FGIDs (n=420) | Infants with FGIDs (n=586) | χ*^2^* | *P* |
| --- | --- | --- | --- | --- | --- |
| Birth weight (kg) (X_1_) | ＜2.5 | 16(3.8) | 23(3.9) | 0.755 | 0.686 |
|  | 2.5～4 | 393(93.6) | 542(92.5) |  |  |
|  | ＞4 | 11(2.6) | 21(3.6) |  |  |
| Parity (X_2_) | ≥2 | 227(54.0) | 309(52.7) | 0.171 | 0.680 |
|  | 1 | 193(46.0) | 277(47.3) |  |  |
| Mode of delivery (X_3_) | Caesarean | 150(35.7) | 202(34.5) | 0.166 | 0.683 |
|  | Natural birth | 270(64.3) | 384(65.5) |  |  |
| Gestational age (week) (X_4_) | ≥42 | 6(1.4) | 2(0.3) | 2.417 | 0.120 |
|  | ＜42 | 414(98.6) | 584(99.7) |  |  |
| Preterm birth (X_5_) | Yes | 8(2.0) | 8(1.4) | 0.455 | 0.500 |
|  | No | 412(98.1) | 578(98.6) |  |  |
| Gender (X_6_) | Male | 236(56.2) | 313(53.4) | 0.761 | 0.383 |
|  | Female | 184(43.8) | 273(46.6) |  |  |
| Native place (X_7_) | Permanent | 207(49.3) | 245(41.8) | 6.015 | **0.049** |
|  | Temporary | 87(20.7) | 129(22.0) |  |  |
|  | Floating | 126(30.0) | 212(36.2) |  |  |
| Rental Housing (X_8_) | Yes | 174(41.4) | 275(46.9) | 2.995 | 0.084 |
|  | No | 246(58.6) | 311(53.1) |  |  |
| Travel mode (X_9_) | private car | 344(81.9) | 459(78.3) | 1.944 | 0.163 |
|  | Others | 76(18.1) | 127(21.7) |  |  |
| Family members (X_10_) | ≤3 | 96(22.9) | 126(21.6) | 1.913 | 0.384 |
|  | 4～6 | 292(69.5) | 418(71.3) |  |  |
|  | ＞6 | 32(7.6) | 42(7.2) |  |  |
| Family Smoking (X_11_) | Yes | 123(29.3) | 145(24.7) | 2.582 | 0.108 |
|  | No | 297(29.3) | 441(75.3) |  |  |
| Father's age (years) (X_12_) | ＜25 | 105(25.0) | 191(32.6) | 6.794 | **0.009** |
|  | ≥25 | 315(75.0) | 395(67.4) |  |  |
| Father's education level (X_13_) | College degree | 333(79.3) | 477(81.4) | 2.182 | 0.336 |
|  | Bachelor’s degree or above | 87(20.7) | 109(18.6) |  |  |
| Father's job stability (X_14_) | Yes | 231(55.0) | 309(52.7) | 0.507 | 0.477 |
|  | No | 189(45.0) | 277(47.3) |  |  |
| Father’s history of FGIDs (X_15_) | Yes | 65(15.5) | 169(28.9) | 24.623 | **0.000** |
|  | No | 355(84.5) | 417(71.2) |  |  |
| Father's smoking history (X_16_) | Yes | 212(50.5) | 292(49.8) | 0.041 | 0.840 |
|  | No | 208(49.5) | 294(50.2) |  |  |
| Mother's age (X_17_) | ＜25 | 64(15.2) | 106(18.1) | 1.416 | 0.234 |
|  | ≥25 | 356(84.8) | 480(81.9) |  |  |
| Mother's education level (X_18_) | College degree | 329(78.3) | 481(82.1) | 5.065 | 0.079 |
|  | Bachelor’s degree or above | 91(21.7) | 105(17.9) |  |  |
| Mother's job stability (X_19_) | Yes | 141(33.6) | 180(30.7) | 0.918 | 0.338 |
|  | No | 279(66.4) | 406(69.3) |  |  |
| Mother’s history of FGIDs (X_20_) | Yes | 67(16.0) | 138(23.5) | 8.597 | **0.003** |
|  | No | 353(84) | 448(76.5) |  |  |
| Fast food taking during pregnancy (X_21_) | Yes | 19(4.5) | 23(3.9) | 0.219 | 0.640 |
|  | No | 401(95.5) | 563(96.1) |  |  |
| Dietary restriction during pregnancy (X_22_) | Yes | 51(12.1) | 72(12.3) | 0.005 | 0.945 |
|  | No | 369(87.9) | 514(87.7) |  |  |
| Unbalanced diet during pregnancy (X_23_) | Yes | 49(11.7) | 81(13.8) | 1.011 | 0.315 |
|  | No | 371(88.3) | 505(86.2) |  |  |
| Disease history during pregnancy (X_24_) | Yes | 105(25.0) | 181(30.9) | 4.168 | **0.041** |
|  | No | 315(75.0) | 405(69.1) |  |  |
| Vit D supplementation during pregnancy (X_25_) | Yes | 24(5.7) | 24(4.1) | 1.411 | 0.235 |
|  | No | 396(94.3) | 562(95.9) |  |  |
| Fast food taking after delivery (X_26_) | Yes | 8(1.9) | 4(0.7) | 3.101 | 0.078 |
|  | No | 412(98.1) | 582(99.3) |  |  |
| Dietary restriction after delivery (X_27_) | Yes | 26(6.2) | 54(9.2) | 3.057 | 0.080 |
|  | No | 394(93.8) | 532(90.8) |  |  |
| Unbalanced diet after delivery (X_28_) | Yes | 19(4.5) | 23(3.9) | 0.219 | 0.640 |
|  | No | 401(95.5) | 563(96.1) |  |  |
| Vit D supplementation after delivery (X_29_) | Yes | 122(29.0) | 163(27.8) | 0.183 | 0.669 |
|  | No | 298(71.0) | 423(72.2) |  |  |
| Vit D supplementation of infants after birth (X_30_) | Yes | 371(88.3) | 479(81.7) | 8.116 | **0.004** |
|  | No | 49(11.7) | 107(18.3) |  |  |
| Calcium supplementation of infants after birth (X_31_) | Yes | 109(26.0) | 124(21.2) | 3.157 | 0.076 |
|  | No | 311(74.0) | 462(78.8) |  |  |
| Probiotic supplementation of infants after birth (X_32_) | Yes | 103(24.5) | 177(30.2) | 3.931 | **0.047** |
|  | No | 317(75.5) | 409(69.8) |  |  |
| Exclusive breastfeeding (X_33_)^a^ | Yes | 231(55.0) | 335(57.2) | 0.467 | 0.494 |
|  | No | 189(45.0) | 251(42.8) |  |  |

^a^ Exclusive breastfeeding means exclusive breastfeeding for 6 months after an infant is born
