# Supplementary material for: A population-based study on prevalence and predisposing risk factors of infant functional gastrointestinal disorders in a single center in Southern Fujian
Source: Front Pediatr. 2022 Sep 29;10:993032. doi: 10.3389/fped.2022.993032 (PMC9557738; doi:10.3389/fped.2022.993032)
Supplement: Supplementary file 3 [file Data_Sheet_1.doc]

**Appendix 1: Questionnaire for** **enrolled infants**

**Part 1. Questionnaire on functional gastrointestinal disorders of infants in Jinjiang city, Fujian province**

Serial number　　　　　　 Follow-up date

Name　　　　　 Date of birth　　　　　　 Birth place

Father’ name　　　　　　 Mother’ name　　　　　 Telephone number

**A．Infant regurgitation**

A1＿＿(0, none; 1, yes)

A2＿＿(times / day)

A3＿＿(when does the disorder begin after birth / day)

A4＿＿(0, partial; 1, all)，A5 the time of duration＿＿(1, less than 7 days; 2, others)

**B. Crying / infant colic**

B1＿＿(0, none; 1, yes)

B2＿＿(cumulative crying time per day, hour)

B3＿＿(when does the disorders begin to occur after birth)

B4＿＿(cumulative times of crying per day)

B5＿＿(frequent period of crying / colic：1, nighttime; 2, daytime), B6 time of duration＿＿(1, couples of days; 2, still so far)

**C. Difficult defecation**

C1＿＿(0, none; 1, yes)

C2＿＿(defecation interval, day)

C3＿＿(0, no measures applied to promote defecation; 1, massage/ Enema Glycerini)

C4＿＿(when does the disorder begin to occur after birth)

C5＿＿(Fecal consistency: normal,0; pasty, 1; liquid, 2; Bristol score: Ⅳ，Ⅴ，Ⅵ，Ⅶ), C6 time of duration＿＿ (1, less than 3 days; 2, others)

**D. Diarrhea**

D1＿＿(0, none; 1, yes)

D2＿＿(the times of defecations per week)

D3＿＿(defecation volume: 0, small; 1, large)

D4＿＿(1, mush; 2, watery)

D5＿＿(when does the disorders begin to occur after birth)

D6＿＿(feces with foams; 2, feces with fats), D7 time of duration＿＿(1, couples of days; 2, still so far)

**E. Constipation**

E1＿＿(0, none; 1, yes)

E2＿＿(defecation interval, day)

E3＿＿(0, no measures applied to promote defecation; 1, massage/ Enema Glycerini)

E4＿＿(when does the disorders begin to occur after birth)

E5＿＿(Bristol score: Ⅰ, Ⅱ, Ⅲ), E6 time of duration＿＿(1, couples of days; 2, still so far)

**F. Other manifestations**

F1＿＿(0, none; 1, yes) details＿＿＿＿＿＿＿＿＿＿＿＿＿＿＿＿＿＿＿＿＿＿＿＿

**G. Intervention**

G1＿＿(0, unnoticed; 1, seeing a doctor) Other measures＿＿＿＿＿＿＿＿＿＿＿＿＿

**H. Memorandum**＿＿＿＿＿＿＿＿＿＿＿＿＿＿＿＿＿＿＿＿＿＿＿＿＿＿＿＿＿＿＿＿＿＿＿＿＿＿＿＿＿＿＿＿＿＿＿＿＿＿＿＿＿＿＿＿＿＿＿＿＿＿＿＿＿＿＿＿＿＿＿＿＿＿＿＿＿＿＿＿＿＿＿＿＿＿＿＿＿＿＿＿＿＿＿＿＿＿＿＿＿＿＿＿＿＿＿

**Part 2. Infants’ growth and development indexes**

**The first visit**

Body weight O1＿＿(kg)；body weight evaluation O2＿＿(percentile)；body length O3＿＿(cm)；body length evaluation O4＿＿(percentile)；head circumference O5＿＿(cm)；head circumference evaluation O6＿＿(percentile)；eczema O7＿＿(0, none; 1, mild; 3, moderate/severe).

**6-month-old follow-up**

body weight P1＿＿(kg)；body length P2＿＿(cm)；head circumference P3＿＿(cm)；Hemoglobin P4＿＿(g/L); eosinophil (EO) P5＿＿(10^9^); EO% P6＿＿%；

**12-month-old follow-up**

body weight P7＿＿(kg)；body length P8＿＿(cm)；head circumference P9＿＿(cm)；Hemoglobin P10＿＿(g/L); eosinophil (EO) P11＿＿（10^9^）; EO% P12＿＿%；

Laboratory findings

Stool routine and parasite tests Q1＿＿(0 , normal; 1, abnormal) ; FOBT Q2＿＿(0, negative; 1, weakly positive; 2, positive ).

**Part 3. Questionnaire on risk factors of functional gastrointestinal disorders in infants in Jinjiang City, Fujian province**

Serial number＿＿＿＿Test date＿＿＿＿＿＿＿

Infant name＿＿＿＿date of birth＿＿＿　＿address

Father’ s name　　　　　　 Mother’s name　　　　　 Telephone number

**Infant’s general condition**

Gender I1＿(1, male 1; 0, female )；Age I2＿＿(months)；Parity I3＿；Production I4＿；Gestational week I5＿birth weight I6＿＿；Mode of delivery I7＿＿(0, spontaneous deliver; 1, surgical assisted delivery; 2, cesarean section)；Delivery place I8＿＿(0, hospital; 1, home)。

**Maternal general condition**

Mother's age J1＿＿(years)；Mother's occupation J2＿＿(1, civil servant; 2, public institutions; 3, business; 4, migrant worker; 5, full-time wife);

Mother's education level J3＿＿(0, illiterate;1, high school degree and below; 2, college degree ; 3, bachelor degree; 4, Master degree or above );

Maternal diseases during pregnancy J4＿(0, no;1, yes) details＿＿＿＿＿＿＿＿＿＿＿＿＿＿＿＿＿＿＿＿；Pre-existing diseases J5＿＿(0, no;1, yes) details＿＿＿＿＿；Smoking history J6＿(0, no, 1, occasionally; 2, often)。

**Paternal general condition**

Father's age K1＿＿(years)；Father's occupation K2＿＿(1, civil servant; 2, public institutions; 3, business; 4, migrant worker; 5, jobless);

Father's education level K3＿＿(0, illiterate;1, high school degree and below; 2, college degree ; 3, bachelor degree; 4, Master degree or above );

Pre-existing diseases K4＿(0, no;1, yes) details＿＿＿＿；Smoking history K5＿(0, no; 1, occasionally; 2, often)。

**Other family members’ general condition**

Smoking history K6＿＿(Number of people)；Family population K7＿＿(Number of people)；Family travel mode K8＿＿(0,Walk; 1, bike, 2, private car)；Family residence K9＿＿(0, commercial housing; 1, self-built house; 2, rented house)；

Place of birth L1＿＿(1, Local resident; 2, non-local resident; 3, migrant workers)。

**Infant feeding**

Formula feeding M1＿＿(0, no;1, yes)；Type of infant feeding M2＿＿(0, Breast milk; 1, partially hydrolyzed formula; 2, mixed feeding; 3, common formula; 4, extensively hydrolyzed formula; 5, amino acid formula; 6, preterm formula)；Addition of cod liver oil/VitD3M3 ＿＿(0, no, 1, occasionally; 2, often)；The time of addition of cod liver oil/VitD3 M4＿＿(months)；Probiotic supplementation after birth M5＿＿(0, no;1, yes)；Calcium supplementation after birth M6＿＿(0, no;1, yes)；others M7＿＿(0, no; 1, DHA; 2, bovine colostrum; 3, both)。

**Diet during the peripartum**

Supplements during late gestation N1＿＿(0, no;1, calcium and cod liver oil; 2, folate; 3, both)；Probiotic supplementation during late gestation N2＿＿(0, no;1, yes)；Supplements after the birth of infant N3＿＿(0, no;1, calcium and cod liver oil; 2, folate; 3, both)；

Unbalanced diet during pregnancy N4＿＿(0, no;1, yes)；Avoid intake of certain food during pregnancy N5＿＿(0, no;1, yes)；Fast food taking frequently during pregnancy N6＿＿(0, no;1, yes)；Unbalanced diet after the birth of infant N7＿＿(0, no;1, yes)；Avoid intake of certain food after the infant birth N8＿＿(0, no;1, yes)；Fast food taking frequently after the birth of infant N9＿＿(0, no;1, yes)。

**Parents’ history of FGIDs**

Maternal history of FGIDs N10＿＿(0, no;1, yes); Paternal history of FGIDs N11＿＿(0, no;1, yes)。
